# COVID-19 associated reduction in elective spay-neuter surgeries for dogs and cats

**DOI:** 10.3389/fvets.2022.912893

**Published:** 2022-09-13

**Authors:** Simone D. Guerios, Tenley R. Porcher, Gina Clemmer, Thomas Denagamage, Julie K. Levy

**Affiliations:** ^1^Department of Small Animal Clinical Sciences, College of Veterinary Medicine, University of Florida, Gainesville, FL, United States; ^2^Clinic HQ, Portland, OR, United States; ^3^Department of Large Animal Clinical Sciences, College of Veterinary Medicine, University of Florida, Gainesville, FL, United States; ^4^Maddie's Shelter Medicine Program, College of Veterinary Medicine, University of Florida, Gainesville, FL, United States

**Keywords:** SARS-CoV-2, dog, cat, pet overpopulation, shelter medicine, sterilization, castration, ovariohysterectomy

## Abstract

The rise in subsidized spay-neuter access helped drive the euthanasia of shelter pets in the US from an estimated 13. 5 million in 1973 to 1.5 million in 2019. When the arrival of the COVID-19 pandemic triggered lockdowns beginning in March 2020, many veterinary providers suspended nonessential services such as routine spay-neuter surgeries. The purpose of this study was to determine the impact of the COVID-19 pandemic on the volume of spay-neuter procedures performed by spay-neuter clinics. A retrospective study of patient data from 212 spay-neuter clinics using Clinic HQ practice management software was conducted from January 2019 through December 2021. The clinics collectively performed 1,217,240 surgeries in the pre-COVID baseline year of 2019. A sharp decline in surgeries began in March 2020 (−22%) and reached a nadir in April 2020 (−80%). Surgeries began to increase in May 2020 (−39%), before plateauing in July 2020 (−6%) and remaining slightly below the 2019 baseline in most months through the end of 2021. Compared to 2019, total surgeries decreased 13% to 1,059,388 in 2020 and decreased 3% to 1,184,274 in 2021. In 2020, when clinic disruptions were highest, the impact of the surgery cutbacks varied by geographic region, species, age, and source of animals. Compared with 2019, in 2020 surgeries decreased 17% in the Midwest region, 15% in the Northeast and West, and 11% in the South. Surgeries were reduced 19% in dogs and 10% in cats. When grouped by age, surgeries were reduced by 18% in geriatrics, 14% in adults, and 11% in juveniles. Reductions were similar for females (−14%) and males (−12%) and similar for unowned/organization-owned animals (−14%) and privately owned animals (−12%). In total, 190,818 fewer surgeries were performed by the 212 studied clinics in the 24 months from January 2020 through December 2021 than would be expected had 2019 levels been maintained. If a similar pattern was experienced by other spay/neuter providers in the US, it would suggest there is a deficit of more than 2.7 million spay/neuter surgeries that animal welfare organizations have yet to address.

## Introduction

Overpopulation of cats and dogs is a global concern. Efforts to manage their population and to reduce the number of animals taken in and euthanized by animal shelters have largely focused on surgical sterilization to reduce unintended reproduction (1). In the US, the first clinics focusing on high-volume spay-neuter emerged in the 1970s (2). By the 1990s, pediatric sterilization became widely accepted, shelters adopted “neuter-before-adoption” policies, and training programs were developed to teach efficient surgical techniques in veterinary schools (3–5). The rise in subsidized spay-neuter access helped drive the euthanasia of shelter pets from an estimated 13.5 million in 1973 (6) to 1.5 million in 2019 (7).

The Association of Shelter Veterinarians defined high-quality, high-volume spay-neuter (HQHVSN) as “efficient surgical initiatives that meet or exceed veterinary medical standards of care in providing accessible, targeted sterilization of large numbers of cats and dogs to reduce their overpopulation and subsequent euthanasia” (8). Mentorship programs and grants were developed to spawn hundreds of clinics specializing in HQHVSN across the US (9). As a high percentage of owned pets were sterilized, attention turned to unowned free-roaming community cats as the primary source of kitten births, inciting trap-neuter-return (TNR) programs to manage their population (10, 11).

In January 2020, the novel SARS-CoV-2 virus reached the US and quickly spread from state to state. The federal government declared a public health emergency in February followed by a national emergency the next month. This triggered states to institute lockdowns beginning in March 2020 that closed schools, businesses, and non-essential public services intended to reduce viral transmission and to preserve medical supplies and personal protective equipment (PPE) for use by healthcare providers. Like their human healthcare counterparts (12, 13), veterinary associations developed guidelines for triaging veterinary care during the pandemic lockdown. While there was no standardized list of essential procedures in veterinary medicine, they were generally grouped into procedures needed to relieve animal pain and suffering, to prevent imminent death or deterioration, and to protect public health (14–16).

While veterinary providers continued to focus on life-threatening emergencies, such as pyometra and dystocia, they largely suspended elective procedures such as preventive care, treatment of minor or chronic conditions, and routine spay-neuter surgeries. Routine spay-neuter surgeries traditionally performed daily at animal shelters and HQHVSN clinics were deemed non-essential. The increased use of telehealth as a substitute for seeing animal patients in person alleviated some veterinary needs during lockdown but could not address the growing gap in spay-neuter services (17–19).

The severe and prolonged pandemic-related decrease in spay-neuter services may have the potential to undermine progress made in controlling pet populations and euthanasia in shelters. The purpose of this study was to examine the impact of the COVID-19 pandemic on the number of spay-neuter procedures performed by HQHVSN clinics in the US.

## Materials and methods

### Data collection

Spay-neuter data for dogs and cats were collected by Clinic HQ (Clinic HQ Inc, Portland, OR), a cloud-based clinic management software program designed for practices focusing on spay-neuter and preventive healthcare services from January 1, 2019 to December 31, 2021. A total of 400 spay-neuter clinics in the United States used the software, which the company estimates included 13% of the approximately 3,000 spay-neuter practices in the US. Of these, 188 were excluded from the study because they did not use the software for the entire baseline year of 2019. Comprehensive patient datasets collected from the remaining 212 clinics from January 2019 to December 2021 were included in this study. This period spanned the 2019 pre-pandemic baseline year of normal operations, the spring 2020 pandemic lockdown during which non-essential services were curtailed, and the following months as restrictions lifted across the country. All clinics were identified by a unique identification number, and the information was collected and stored in an electronic format. No identifiable client, patient or clinic information was stored with the data available for the study.

The data were categorized for both dog and cat populations for the following variables: sex (male vs. female), age (pediatric <5 months, adult 5 months to 7 years, and geriatric > 7 years), and ownership status. Ownership status included privately owned pets vs. unowned pets (community cats, pets from municipal and private shelters, and rescue organizations). Four geographic regions were used for analysis, based on the US census bureau regions (20): Northeast (Connecticut, Maine, Massachusetts, New Hampshire, New Jersey, New York, Pennsylvania, Rhode Island, and Vermont), Midwest (Indiana, Illinois, Michigan, Ohio, Wisconsin, Iowa, Kansas, Minnesota, Missouri, Nebraska, South Dakota, and North Dakota), South (Delaware, District of Colombia, Florida, Georgia, Maryland, North Carolina, South Carolina, Virginia, West Virginia, Alabama, Kentucky, Mississippi, Tennessee, Arkansas, Louisiana, Oklahoma, and Texas), and West (Arizona, Colorado, Idaho, New Mexico, Montana, Utah, Nevada, Wyoming, Alaska, California, Hawaii, Oregon, and Washington).

### Descriptive statistics

Data from 212 clinics from January 1, 2019 to December 31, 2021 were included in the analysis. The number of total surgeries performed each year and the absolute and percentage change from 2019 were determined for month, species, sex, age, ownership status, and geographical region.

## Results

From a total of 400 spay-neuter clinics that currently used the software, 212 had complete spay-neuter surgery data for the entire 3-year study period. These clinics collectively performed 1,217,240 spay-neuter surgeries in the baseline year of 2019 (Table 1). Surgeries decreased 13% to 1,059,388 in 2020, then rebounded to 1,184,274 in 2021, still 3% below the baseline year. By December 2021, 8 clinics (4%) had temporarily closures, 4 clinics (2%) were permanently closed, and 2 clinics (1%) stopped performing spay-neuter surgeries and converted to wellness services only. In total, 190,818 fewer surgeries were performed by the 212 studied clinics in the 24 months from January 2020 through December 2021 than would be expected had 2019 levels been maintained.

**Table 1:** Number of cats and dogs spayed or neutered, absolute change, and percentage change by month from January 2019 through December 2021.

**Month**	**2019**	**2020**	**2021**	**Absolute (%) change 2019 vs. 2020**	**Absolute (%) change 2019 vs. 2021**
January	101,565	106,226	97,114	+4,661 (+5)	−4,451 (−4)
February	93,216	97,340	91,511	+4,124 (+4)	−1,705 (−2)
March	101,779	79,089	111,257	−22,690 (−22)	+9,478 (+9)
April	101,151	19,818	101,432	−81,333 (−80)	+281 (0)
May	98,455	60,460	93,379	−37,995 (−39)	−5,076 (−5)
June	98,549	95,106	106,805	−3,443 (−3)	+8,256 (+8)
July	107,727	101,755	100,319	−5,972 (−6)	−7,408 (−7)
August	111,784	101,519	102,368	−10,265 (−9)	−9,416 (−8)
September	103,245	104,780	98,376	+1,535 (+1)	−4,869 (−5)
October	119,622	110,439	99,271	−9,183 (−8)	−20,351 (−17)
November	96,434	93,080	96,826	−3,354 (−3)	+392 (0)
December	83,713	89,776	85,616	+6,063 (+7)	+1,903 (+2)
*Total*	*1,217,240*	*1,059,388*	*1,184,274*	*−157,852 (−13)*	*−32,966 (−3)*

**Figure 1:**
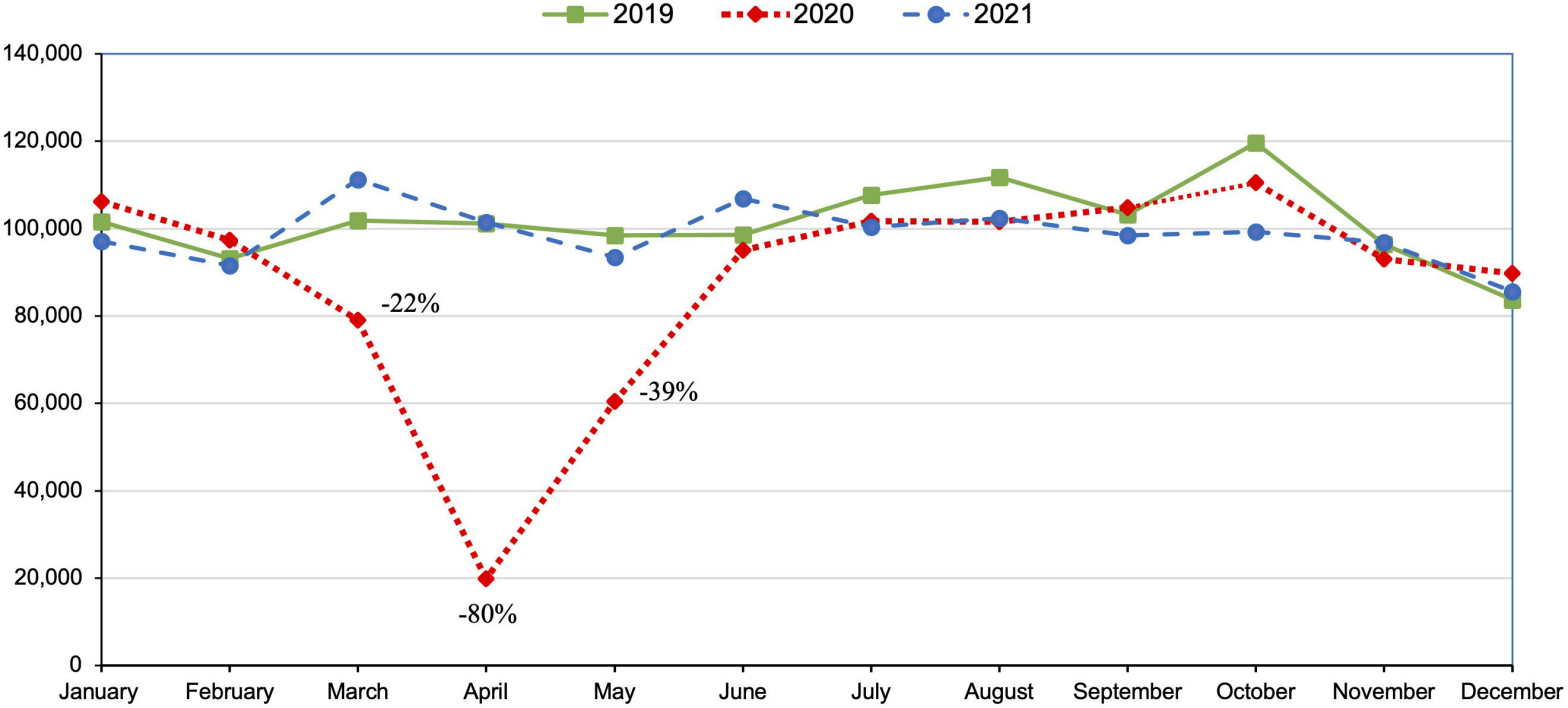
Number of cats and dogs spayed or neutered per month at 212 spay-neuter clinics in the US during baseline year 2019, and COVID-19 pandemic years 2020 and 2021.

Total surgeries per month were compared for the entire study period (Figure 1 and Table 1). The clinics started 2020 with 5% more surgeries in January and 4% in February than during the same period in 2019. A sharp decline in surgeries began in March 2020 (−22%) and reached a nadir in April 2020 (−80%) compared to the baseline year of 2019. Surgeries began to increase in May 2020 (−39%), before plateauing in June 2020 (−3%) and remaining slightly below baseline in most months throughout the rest of the study period (−17% to +9%).

Trends in the spay-neuter data for dogs and cats were reported as the total number of animals per year, absolute change from 2019, and percentage change from 2019 (Table 2) (Supplementary Tables I, II). In 2019, a majority of surgeries were performed in cats (66%) vs. dogs (34%), a pattern that remained consistent throughout the study period. Total surgeries decreased in both dogs (−19% in 2020; −14% in 2021) and cats (−10% in 2020; +3% in 2021). A majority of surgeries in 2019 were performed in females (53%) vs. males (47%), and this pattern persisted throughout the study period. Total surgeries decreased in both females (−14% in 2020; −4% in 2021) and males (−12% in 2020; −1% in 2021).

**Table 2:** Number of cats and dogs spayed or neutered, by species, sex, age, ownership status, and US regions in 2020 and 2021 compared to the baseline year of 2019.

**Variable**	**Category**	**2019**	**2020**	**2021**	**Absolute (%) change 2019 vs. 2020**	**Absolute (%) change 2019 vs. 2021**
Species	Cat	806,886	728,555	832,688	−78,330 (−10)	+25,802 (+3)
	Dog	410,354	330,833	351,586	−79,520 (−19)	−58,768 (−14)
Sex	Male	568,727	498,899	560,915	−68,828 (−12)	−7,812 (−1)
	Female	648,513	560,489	623,359	−88,024 (−14)	−25,154 (−4)
Agey^a^	Pediatric	362,244	321,419	333,654	−40,825 (−11)	−28,590 (−8)
	Adult	843,896	728,833	841,258	−115,063 (−14)	−2,638 (0)
	Geriatric	11,100	9,136	9,362	−1,964 (−18)	−1,738 (−16)
Ownership^b^	Owned	764,517	671,746	754,360	−92,771 (−12)	−10,157 (−1)
	Unowned	452,723	387,642	429,914	−65,081 (−14)	−22,809 (−5)
US Region	South	565,689	505,734	552,278	−59,955 (−11)	−13,411 (−2)
	Midwest	104,515	87,066	96,346	−17,449 (−17)	−8,169 (−8)
	West	288,665	245,821	295,824	−42,844 (−15)	+7,159 (+2)
	Northeast	258,371	220,767	239,826	−37,605 (−15)	−18,545 (−7)
*Total*		*1,217,240*	*1,059,388*	*1,184,274*	*−157,852* (−13)	*−32,966* (−3)

a Pediatric (<5 months), Adult (5 months−7 years), Geriatric (>7 years).

b Owned (privately owned pets), Unowned (community cats and pets from municipal and private shelters and rescue organizations).

Adult-aged animals (5 months to 7 years) were the largest group in 2019 (69%), followed by pediatric animals (<5 months) (30%) and geriatric animals (more than 7 years) (1%). Total surgeries decreased in geriatrics (−18% in 2020; −16% 2021), adults (−14% in 2020; 0% in 2021), and pediatrics (−11% in 2020; −8% in 2021). A majority of surgeries were performed in owned animals (63%) vs. unowned animals (37%), a pattern that remained consistent throughout the study. Total surgeries decreased in both owned animals (−12% in 2020; −1% in 2021) and in unowned animals (−14% in 2020; −5% in 2021).

Clinics in the South region performed the highest proportion of total surgeries in 2019 (47%), followed by West (23%), Northeast (21%) and Midwest (9%). Total surgeries decreased in the Midwest (−17% in 2020; −8% in 2021), the Northeast (−15% in 2020; −7% in 2021) and West (−15% in 2020; +2% in 2021), and the South (−11% in 2020; −2% in 2021).

## Discussion

The COVID-19 pandemic led to a sudden and large reduction in the number of spay-neuter surgeries performed by spay-neuter clinics in March and April 2020. Surgeries began to rebound in May 2020, coinciding with the development of safety measures such as masking, physical distancing, testing, and curbside veterinary reception. In total, 190,818 fewer surgeries were performed by the 212 studied clinics in the 24 months from January 2020 through December 2021 than would be expected had 2019 levels been maintained. If a similar pattern was experienced by all 3,000 estimated spay-neuter clinics in the US, it would suggest a deficit of more than 2.7 million spay-neuter surgeries have accumulated by the end of 2021. The decrease in elective spay-neuter procedures observed in this study echoed a similar decline in elective surgical procedures in human medicine during the 7 weeks after the initial lockdown, a return to near 2019 numbers in the following months, and a deficit in total number of surgeries performed by the end of the first year of the pandemic (21). Since pandemic conditions have not yet resolved as of August 2022, spay-neuter clinics have not yet returned to baseline productivity, and an ongoing workforce shortage of veterinarians and staff threatens the spay-neuter recovery, conditions are ripe for an increase in unwanted litters and a reversal of population control gains of the past decade (22). Veterinary and animal welfare organizations should prioritize recovery of spay-neuter capacity and focus on populations most at risk for unintended reproduction, poor welfare, or entering the animal shelter system.

Clinics performed nearly twice as many feline vs. canine surgeries in the baseline year, and reduced canine surgeries almost twice as much as feline surgeries during the pandemic. While it is unknown why this cohort of clinics experienced this pattern, it could be related to the urgency associated with cats being euthanized at twice the rate of dogs in shelters, the proliferation of TNR programs for community cats, and the ease of performing cat surgery while simultaneously maintaining physical distancing (17, 23). Cat reproduction is highly seasonal, peaking in spring and summer. The delayed return of full capacity spay-neuter clinics has now spanned three “kitten seasons,” causing concern about population growth of free-roaming community cats (24).

Geriatric animals constituted the smallest age bracket in the baseline year and had the greatest reduction in surgery during the pandemic. Geriatric animals often require greater preparation for surgery, for example pre-surgical laboratory testing, and more intensive monitoring. This, combined with the fact that geriatric animals are at lower risk for unwanted reproduction, may explain the decrease in surgery for older animals (25, 26). The decrease in surgery was less marked for younger animals, with adults remaining the largest age group to have surgery throughout the study period. Adult animals are at immediate risk for reproduction, whereas juveniles would reach reproductive age after just a few months. Clinics performed a similar reduction in spays compared to neuters during the pandemic, even though female sterilization is most important for preventing unintended reproduction. The prolonged delay in return to normal spay-neuter operations is especially concerning for female cats as they can become pregnant as early of 5 months of age, which underpins the recommendation that cats be sterilized before 5 months of age (27).

Unowned pets (community cats and pets from shelters and rescue organizations) experienced a similar decrease in spay-neuter surgeries compared to privately owned pets. This may be related to the completion of pre-existing surgery appointments for owned pets as well as reduced numbers of animals held in shelters. Many animal shelters suspended “neuter-before-adoption” policies in order to quickly move shelter pets into permanent or foster homes as they sought to reduce shelter pet populations during the transition to essential services only (28, 29). Alternatives included releasing pets with contracts or vouchers to have them sterilized when spay-neuter capacity resumed or placement with “foster-to-adopt” agreements in which ownership was not transferred until documentation of sterilization was provided (30). In addition, the National Animal Care and Control Association (NACA) advised suspension of trapping and intaking healthy community cats or other animals not in immediate danger during the pandemic (29, 31).

The decrease in surgery coincided with the high number of COVID-19 cases early in the pandemic. In April 2020, there were nearly 400,000 reported cases of SARS-CoV-2 infection in the US. Of these, 54% were from states in the Northeast, leading to stricter business lockdowns in this region (32). The Midwest, West, and Northeast regions of the US had the largest proportional decline in spay-neuter surgeries. Pet population numbers in the Northeast in particular are generally in balance with demand for pets (6, 33), shelters are not overcrowded, and the per capita demand for HQHVSN services is lower than in other regions where pet overpopulation persists. In contrast, pet overpopulation, shelter crowding, and euthanasia for population control is highest in the South, where spay-neuter activity remains a high priority. Clinics in the South collectively performed the most surgeries before and during the pandemic and experienced the smallest proportional decline in surgeries during the pandemic. COVID-19 restrictions and mandates were lifted at different times for each state, which affected when shelters and clinics could begin returning to normal operations. Northeast states such as Connecticut, lifted most business restrictions in May 2021, while Southern states were less restricted throughout the pandemic (34). The future impact of the spay-neuter gap across regions is likely to be complex as it is influenced not just by the number of surgeries, but also by factors such as mild climates that support greater survival of offspring, the pre-existing number of free-roaming animals, particularly community cats, and regional access to low-cost veterinary care in underserved communities.

Guidelines specifically aimed at safe practices in spay-neuter clinics became available soon after the initial pandemic lockdown (24, 35). However, as the pandemic became prolonged, providers experienced staffing shortages associated with an ongoing veterinary workforce shortage compounded by the impact of the pandemic depleting staffing numbers due to SARS-CoV-2 infections, quarantines, and the need to stay home with children and other dependents (36). These staffing shortages threaten to undermine the recovery of HQHVSN programs and other animal welfare initiatives. When spay-neuter capacity is limited, providers can prioritize patients based on both safety and urgency (35, 37). When COVID activity is high and physical distancing is important, selecting animals and procedures that reduce personnel contact reduces the risk of workplace transmission (14). For example, use of intramuscular anesthetic induction protocols and selection of smaller patients, such as puppies, cats, and small breed dogs, make it possible for a single staff member to prepare a patient for surgery. Surgery and recovery times are shorter in juveniles and small adults, enabling more surgeries to be completed when capacity is limited (38). In addition to treating reproductive emergencies, providers can prioritize animals most at risk for unintended reproduction, such as pregnant animals, mixed sex litters, females, and community cats, as well as animals at risk for behavior issues such as spraying by intact male cats.

Limitations of this study include the use of a single baseline year, whereas a longer time would have provided more information about temporal trends of spay-neuter surgeries prior to the pandemic. Focusing exclusively on clinics utilizing the Clinic HQ spay-neuter specialty software excluded evaluation of surgery trends in full-service practices and organizations utilizing other veterinary management software programs. However, this software is widely used in the HQHVSN industry. Given the size of the dataset and the broad geographical representation, the authors believe it is illustrative of spay-neuter trends across the US. Although significant differences as a function of species, age, ownership, and region were observed, the study design did not provide a means to determine the reason for the differences, whether they were intentional, or to what extent lockdowns, funding, and/or staffing played a role.

## Conclusion

At the beginning of 2020, a cohort of 212 spay-neuter clinics that collectively performed more than 1 million surgeries per year were on track to increase surgeries by 5% over the previous year. The COVID-19 pandemic lockdown resulted in a drastic reduction of surgeries, from which the industry has not yet recovered. The high level of spay-neuter achieved over the past five decades is the single most important driver of reduced pet overpopulation and euthanasia in animal shelters. Veterinarians and animal welfare organizations should collaborate to prioritize recovery of spay-neuter capacity with a special focus on serving populations most at risk for unintended reproduction, poor welfare, or entering the animal shelter system.

## Data availability statement

The raw data supporting the conclusions of this article will be made available by the authors, without undue reservation.

## Author contributions

JL and GC conceptualized the research topic. GC, SG, and TP compiled the data sets. TD performed statistical analysis. SG, TP, and JL prepared the manuscript. All authors contributed to the article and approved the submitted version.

## Funding

This work was supported by a summer research grant provided through the Boehringer Ingelheim Veterinary Scholars Program and the Department of Small Animal Clinical Sciences at the University of Florida College of Veterinary Medicine.

## Conflict of interest

The authors declare that the research was conducted in the absence of any commercial or financial relationships that could be construed as a potential conflict of interest.

## Publisher's note

All claims expressed in this article are solely those of the authors and do not necessarily represent those of their affiliated organizations, or those of the publisher, the editors and the reviewers. Any product that may be evaluated in this article, or claim that may be made by its manufacturer, is not guaranteed or endorsed by the publisher.

## References

[1] Hiby E Atema KN Brimley R Hammond-Seaman A Jones M Rowan A. Scoping review of indicators and methods of measurement used to evaluate the impact of dog population management interventions. BMC Vet Res. (2017) 13:143. 10.1186/s12917-017-1051-2 28558736 PMC5450220

[2] Collins TF. Control of pet animals. S Afr Med J. (1976) 50:1054–7. 951628

[3] Gates MC Littlewood KE Kongara K Odom TF Sawicki RK. Experience of practicing veterinarians with supervising final-year students and new graduates in performing desexing surgeries. J Vet Med Educ. (2020) 47:465–74. 10.3138/jvme.0918-100r 32412365

[4] Shivley JM Brookshire WC Bushby PA Woodruff KA. Clinically prepared veterinary students: enhancing veterinary student hands-on experiences and supporting hospital caseload using shelter medicine program. Front Vet Sci. (2018) 5:95. 10.3389/fvets.2018.00095 29868617 PMC5958676

[5] Rigdon-Brestle K Accornero VH Amtower M Slater MR. Retrospective review reveals few complications of ovarian pedicle tie in 15,927 cats undergoing ovariohysterectomy at a large HQHVSN clinic and training facility in the United States: 2017-2018. J Am Vet Med Assoc. (2022) 1–8. 10.2460/javma.21.09.0405 [Epub ahead of print]. 35333751

[6] Rowan A Kartal T. Dog population & dog sheltering trends in the United States of America. Animals. (2018) 8:68. 10.3390/ani8050068 29710771 PMC5981279

[7] ASPCA. How Many Pets Are in the United States? How Many Animals Are in Shelters? (2019). Available online at: https://www.aspca.org/helping-people-pets/shelter-intake-and-surrender/pet-statistics (accessed June 13, 2021).

[8] Griffin B Bushby PA McCobb E White SC Rigdon-Brestle YK Appel LD. The Association of shelter veterinarians' 2016 veterinary medical care guidelines for spay-neuter programs. J Am Vet Med Assoc. (2016) 249:165–88. 10.2460/javma.249.2.165 27379593

[9] ASPCApro. Ten Million Cats and Dogs Sterilized Through ASPCA Spay/Neuter Alliance Mentorship. (2020). Available online at: https://www.aspcapro.org/news/2020/02/24/ten-million-cats-and-dogs-sterilized-through-aspca-spayneuter-alliance-mentorship (accessed March 22, 2022).

[10] Levy JK Isaza NM Scott KC. Effect of high-impact targeted trap-neuter-return and adoption of community cats on cat intake to a shelter. The Vet J. (2014) 201:269–74. 10.1016/j.tvjl.2014.05.001 24980808

[11] Spehar DD Wolf PJ. Integrated return-to-field and targeted trap-neuter-vaccinate-return programs result in reductions of feline intake and euthanasia at six municipal animal shelters. Front Vet Sci. (2019) 6:77. 10.3389/fvets.2019.00077 30949486 PMC6437086

[12] Giuffrida M Cozzani F Rossini M Bonati E Del Rio P. How COVID-19 pandemic has changed elective surgery: the experience in a general surgery unit at a COVID-hospital. Acta Biomed. (2021) 92:e2021304. 10.23750/abm.v92i5.10296 34738588 PMC8689307

[13] Diaz A Sarac BA Schoenbrunner AR Janis JE Pawlik TM. Elective surgery in the time of COVID-19. Am J Surg. (2020) 219:900–2. 10.1016/j.amjsurg.2020.04.014 32312477 PMC7161516

[14] Dalton KR Guyer KM Schiaffino F Ferradas C Falke JR Beasley EA. Assessing covid-19 pandemic risk perception and response preparedness in veterinary and animal care workers. Health Secur. (2022) 20:116–26. 10.1089/hs.2021.0091 35108121 PMC9081026

[15] Weese S. Veterinary Medicine During a Time of Restriction of Elective Services Social Distancing. Ontario Veterinary Medical Association (2020). ON: WormSandegerms. Available online at: https://www.wormsandgermsblog.com/files/2020/04/COVID-guidelines-essential-elective_social-distancing_April-17.pdf (accessed April 2, 2022).

[16] Stavisky J Mugford A Deam R Taylor S Barfield D Borgeat K. Dog Cat Triage Guidance for Use During COVID-19 Pandemic in the United Kingdom. BSVA (2020). p. 1–26. Available online at: https://www.researchgate.net/publication/340273440_Dog_and_cat_triage_guidance_for_use_during_COVID-19_pandemic_BSAVA_guidance_document(accessed March 22, 2022).

[17] Kogan LR Erdman P Currin-McCulloch J Bussolari C Packman W. The impact of covid on cat guardians: Vet Iss. Animals. (2021) 11:603. 10.3390/ani11030603 33668841 PMC7996145

[18] Hoffman CL. The experience of teleworking with dogs and cats in the united states during COVID-19. Animals. (2021) 11:268. 10.3390/ani11020268 33494484 PMC7912221

[19] Association AVM. COVID-19 Impact on Veterinary Practices. (2020). Available online at: https://www.avma.org/resources-tools/animal-health-and-welfare/covid-19/covid-19-impact-veterinary-practices (accessed January 12, 2022).

[20] Bureau USC. Census Regions and Divisions of the United States (2010). Available online at: https://www2.census.gov/geo/pdfs/maps-data/maps/reference/us_regdiv.pdf (accessed June 13, 2021).

[21] Mattingly AS Rose L Eddington HS Trickey AW Cullen MR Morris AM. Trends in US surgical procedures and health care system response to policies curtailing elective surgical operations during the COVID-19 pandemic. JAMA Netw Open. (2021) 4:e2138038. 10.1001/jamanetworkopen.2021.38038 34878546 PMC8655602

[22] Gortázar C de la Fuente J. COVID-19 is likely to impact animal health. Prev Vet Med. (2020) 180:105030. 10.1016/j.prevetmed.2020.105030 32447153 PMC7255270

[23] Aeluro S Buchanan JM Boone JD Rabinowitz PM. “State of the mewnion”: practices of feral cat care and advocacy organizations in the United States. Front Vet Sci. (2021) 8:791134. 10.3389/fvets.2021.791134 34970620 PMC8712445

[24] ASPCApro. Statement of Support for Veterinarians Considering Megestrol Acetate as a Temporary Contraceptive for Female Cats During COVID-19. (2021). Available online at: https://www.aspcapro.org/resource/aspca-statement-support-veterinarians-considering-megestrol-acetate-temporary (accessed March 22, 2022).

[25] Hawes SM Kerrigan JM Hupe T Morris KN. Factors informing the return of adopted dogs and cats to an animal shelter. Animals. (2020) 10:1573. 10.3390/ani10091573 32899419 PMC7552273

[26] Hawes S Kerrigan J Morris K. Factors informing outcomes for older cats and dogs in animal shelters. Animals. (2018) 8:36. 10.3390/ani8030036 29518897 PMC5867524

[27] Bushby PA. High-quality, high-volume spay-neuter: access to care and the challenge to private practitioners. J Feline Med Surg. (2020) 22:208–15. 10.1177/1098612X20903600 32093579 PMC11132592

[28] ASPCApro. Statement Concerning Spay and Neuter of Shelter Animals During the COVID-19 Crisis. (2021). Available online at: https://www.aspcapro.org/resource/aspca-statement-concerning-spay-and-neuter-shelter-animals-during-covid-19-crisis (accessed January 12, 2022).

[29] Association NACC,. Statement on Releasing Unaltered Pets From Animal Shelters During the COVID-19 Pandemic. (2020). Available online at: https://www.nacanet.org/wp-content/uploads/2020/03/3.Releasing-Unaltered-Pets-from-Animal-Shelters-During-the-COVID-19-Pandemic.pdf (accessed March 22, 2022).

[30] Hoffman CL Thibault M Hong J. Characterizing pet acquisition and retention during the COVID-19 pandemic. Front Vet Sci. (2021) 8:781403. 10.3389/fvets.2021.781403 34869749 PMC8637628

[31] Association NACC. Statement on Cat Intake Protocol Recommendations During COVID-19 Pandemic. (2020). Available online at: https://www.nacanet.org/wp-content/uploads/2020/04/2.Cat-Intake-Protocols-Statement-1.pdf (accessed January 12, 2022).

[32] Prevention CDC. Geographical Differences in COVID-19 Cases, Death, and Incidences - United States, February 12-April 7, 2020. (2020). 10.15585/mmwr.mm6915e4 PMC7755058 32298250

[33] Woodruff K Smith DR. An estimate of the number of dogs in us shelters in 2015 and the factors affecting their fate. J Appl Anim Welf Sci. (2020) 23:302–14. 10.1080/10888705.2019.1663735 31488001

[34] AARP. List of Coronavirus-Related Restriction in Every State. (2022). Available online at: https://www.aarp.org/politics-society/government-elections/info-2020/coronavirus-state-restrictions.html (accessed March 22, 2022).

[35] ASPCApro. COVID-19 Spay/Neuter and Wellness Clinic Preparedness Guide. (2020). Available online at: https://www.aspcapro.org/resource/covid-19-spayneuter-and-wellness-clinic-preparedness-guide (accessed March 22, 2022).

[36] News VP,. Staffing Shortage Threatens Health of 75 Million Pets by 2030. (2020). Available online at: https://www.veterinarypracticenews.com/75-million-pets-may-lose-access-to-care-by-2030 (accessed March 22, 2022).

[37] McCallin AJ Hough VA Kreisler RE. Pyometra management practices in the high quality, high volume spay-neuter environment. Top Companion Anim Med. (2021) 42:100499. 10.1016/j.tcam.2020.100499 33249239

[38] Spain CV Scarlett JM Houpt KA. Long-term risks and benefits of early-age gonadectomy in dogs. J Am Vet Med Assoc. (2004) 224:380–7. 10.2460/javma.2004.224.380 14765797

