# Supplementary material for: COVID-19 associated reduction in elective spay-neuter surgeries for dogs and cats
Source: Front Vet Sci. 2022 Sep 13;9:912893. doi: 10.3389/fvets.2022.912893 (PMC9513967; doi:10.3389/fvets.2022.912893)
Supplement: Supplementary file 1 [file Data_Sheet_1.docx]

**Supplementary Table I.** Total number of cats spayed or neutered, by sex, age, ownership status, and US regions in 2020 and 2021 compared to the baseline year of 2019.

| **Variable** | **Category** | **2019** | **2020** | **2021** | **Absolute (%) changes 2020 vs. 2019** | **Absolute (%) changes 2021 vs. 2019** |
| --- | --- | --- | --- | --- | --- | --- |
| **Sex** | Male | 371,484 | 338,708 | 388,555 | -32,776 (-9) | +17,071 (+5) |
|  | Female | 435,402 | 389,847 | 444,133 | -45,555 (-10) | +8,731 (+2) |
|  |  |  |  |  |  |  |
| **Age** ^a^ | Pediatric | 283,843 | 260,375 | 273,452 | -23,468 (-8) | -10,391 (-4) |
|  | Adult | 519,443 | 464,895 | 555,475 | -54,548 (-11) | +36,032 (+7) |
|  | Geriatric | 3,600 | 3,285 | 3,761 | -315 (-9) | +161 (+4) |
|  |  |  |  |  |  |  |
| **Ownership** ^b^ | Owned | 485,696 | 443,817 | 508,394 | -41,879 (-9) | +22,698 (+5) |
|  | Unowned | 321,190 | 284,738 | 324,294 | -36,452 (-11) | 3,104 (+1) |
|  |  |  |  |  |  |  |
| **US Region** | South | 349,464 | 322,015 | 359,847 | -27,449 (-8) | +10,383 (+3) |
|  | Midwest | 83,362 | 72,238 | 79,323 | -11,124 (-13) | -4,039 (-5) |
|  | West | 197,172 | 177,396 | 219,531 | -19,776 (-10) | +22,359 (+11) |
|  | Northeast | 176,888 | 156,906 | 173,987 | -19,982 (-11) | -2,901 (-2) |
| *Total* |  | *806,886* | *728,555* | *832,688* | *-78,331 (-10)* | *+25,802 (3)* |

^a^ Pediatric (< 5 months), Adult (5 months-7 years), Geriatric (> 7 years).

^b^ Owned (privately owned pets), Unowned (community cats and pets from municipal and private shelters and rescue organizations).

**Supplementary Table II.** Number of dogs spayed or neutered, by sex, age, ownership status, and US regions in 2020 and 2021 compared to the baseline year of 2019.

| **Variable** | **Category** | **2019** | **2020** | **2021** | **Absolute (%) changes 2020 vs. 2019** | **Absolute (%) changes 2021 vs. 2019** |
| --- | --- | --- | --- | --- | --- | --- |
| **Sex** | Male | 197,243 | 160,191 | 172,360 | -37,052 (-19) | -24,883 (-13) |
|  | Female | 213,111 | 170,642 | 179,226 | -42,469 (-20) | -33,885 (-16) |
|  |  |  |  |  |  |  |
| **Age** ^a^ | Pediatric | 78,401 | 61,044 | 60,202 | -17,357 (-22) | -18,199 (-23) |
|  | Adult | 324,453 | 263,938 | 285,783 | -60,515 (-19) | -38,670 (-12) |
|  | Geriatric | 7,500 | 5,851 | 5,601 | -1,649 (-22) | -1,899 (-25) |
|  |  |  |  |  |  |  |
| **Ownership** ^b^ | Owned | 278,821 | 227,929 | 245,966 | -50,892 (-18) | -32,855 (-12) |
|  | Unowned | 131,533 | 102,904 | 105,620 | -28,629 (-22) | -25,913 (-20) |
|  |  |  |  |  |  |  |
| **US Region** | South | 216,225 | 183,719 | 192,431 | -32,506 (-15) | -23,794 (-11) |
|  | Midwest | 21,153 | 14,828 | 17,023 | -6,325 (-30) | -4,130 (-20) |
|  | West | 91,493 | 68,425 | 76,293 | -23,068 (-25) | -15,200 (-17) |
|  | Northeast | 81,483 | 63,861 | 65,839 | -17,622 (-22) | -15,644 (-19) |
| *Total* |  | *410,354* | *330,833* | *351,586* | *-79,521* (-19) | *-58,768* (-14) |

^a^ Pediatric (< 5 months), Adult (5 months-7 years), Geriatric (> 7 years).

^b^ Owned (privately owned pets), Unowned (community cats and pets from municipal and private shelters and rescue organizations).
